# *OsGRETCHENHAGEN3-2* modulates rice seed storability via accumulation of abscisic acid and protective substances

**DOI:** 10.1093/plphys/kiab059

**Published:** 2021-02-11

**Authors:** Zhiyang Yuan, Kai Fan, Yuntong Wang, Li Tian, Chaopu Zhang, Wenqiang Sun, Hanzi He, Sibin Yu

**Affiliations:** National Key Laboratory of Crop Genetic Improvement, College of Plant Science and Technology, Huazhong Agricultural University, Wuhan 430070, China

## Abstract

Seed storability largely determines the vigor of seeds during storage and is significant in agriculture and ecology. However, the underlying genetic basis remains unclear. In the present study, we report the cloning and characterization of the rice (*Oryza sativa*) indole-3-acetic acid (IAA)-amido synthetase gene *GRETCHEN HAGEN3-2* (*OsGH3-2*) associated with seed storability. *OsGH3-2* was identified by performing a genome-wide association study in rice germplasms with linkage mapping in chromosome substitution segment lines, contributing to the wide variation of seed viability in the populations after long periods of storage and artificial ageing. *OsGH3-2* was dominantly expressed in the developing seeds and catalyzed IAA conjugation to amino acids, forming inactive auxin. Transgenic overexpression, knockout, and knockdown experiments demonstrated that *OsGH3-2* affected seed storability by regulating the accumulation level of abscisic acid (ABA). Overexpression of *OsGH3-2* significantly decreased seed storability, while knockout or knockdown of the gene enhanced seed storability compared with the wild-type. *OsGH3-2* acted as a negative regulator of seed storability by modulating many genes related to the ABA pathway and probably subsequently late embryogenesis-abundant proteins at the transcription level. These findings shed light on the molecular mechanisms underlying seed storability and will facilitate the improvement of seed vigor by genomic breeding and gene-editing approaches in rice.

## Teaser

Inactivation of indole-3-acetic acid by an IAA-amido synthetase inhibits abscisic acid signaling and reduces protective proteins, leading to poor seed storability in rice.

## Introduction

Seeds play a vital role in the plant life cycle and are of economic importance as starting point at sowing and the endpoint of the harvest in agriculture. Seeds are usually stored to maintain the genetic hardware for crop production seasonally. Seed storability or seed longevity is defined as the ability to remain alive during storage and is a vital trait for agricultural production and germplasm preservation in crops. As the loss of seed viability is caused by deterioration during storage, seed life is limited (Rajjou and Debeaujon, 2008). The optimal storage conditions, such as low relative humidity and low temperature, are conducive to the extension of seed life span. However, these conditions are rather costly to realize and maintain. Genetic improvement is thus considered as the most promising strategy to enhance seed storability and decrease seed storage cost. 

Many cereal crops show substantial variations in seed storability. Such variation usually exhibits a continuous phenotypic distribution controlled by polygenes or quantitative trait loci (QTLs) and is strongly affected by environment during seed formation, harvest, and storage (Bentsink et al., 2000; Miura et al., 2002; Clerkx et al., 2004b; Zuo et al., 2018) . Unraveling the genetic architecture of seed storability is a prerequisite for genetic improvement. Great efforts have been made to dissect the genetic bases underlying seed storability variation through linkage analyses and genome-wide association studies (GWAS) in various model plants and crops, such as Arabidopsis (*Arabidopsis thaliana*; Bentsink et al., 2000; Nguyen et al., 2012), lettuce (*Lactuca sativa*; Schwember and Bradford, 2010), rice (*Oryza sativa*; Miura et al., 2002; Liu et al., 2018), barley (*Hordeum vulgare*; Wozny et al., 2018), and wheat (*Triticum aestivum*; Zuo et al., 2018). In rice, the seed storability of *indica* is generally stronger than that of temperate *japonica* (Rao and Jackson, 1996)*.* Over 60 QTLs associated with seed viability were detected in various rice populations (Miura et al., 2002; Hang et al., 2015; Liu et al., 2018; Lee et al., 2019). However, most QTLs identified for seed storability are different in various genetic populations, and only a few loci have been subjected to a map-based cloning strategy to identify the genes responsible for seed storability. For example, *qSS-9/qLG-9* was mapped as a common QTL for seed storability or seed longevity in several populations (Miura et al., 2002; Lin et al., 2015; Yuan et al., 2019), which was finely mapped to a 30-kb region (Sasaki et al., 2015). Recently, seven loci of seed storability were identified in small regions less than 50 kb using the Nipponbare/9311 backcross inbred line population with a high-density single nucleotide polymorphism (SNP) linkage map. Among them, *OsFAH2* encoding a fatty acid hydroxylase that controls seed storability was cloned (Yuan et al., 2019). These studies provide an opportunity to dissect the molecular mechanisms underlying seed storability in crops.

Plant phytohormones play key roles in regulating seed maturation and the acquisition of storability (Clerkx et al., 2003; Carranco et al., 2010; Bueso et al., 2014; Zinsmeister et al., 2016; Leprince et al., 2017; Sano et al., 2017). Auxin or indole-3-acetic acid (IAA) acts as a versatile trigger in many developmental processes. The *GRETCHEN HAGEN3* (*GH3*) gene family is a key factor in modulating homeostasis of auxin through the conjugation of free IAA to amino acids (Staswick et al., 2005; Woodward and Bartel, 2005; Weijers and Wagner, 2016; Leyser, 2018). The rice *GH3* gene family includes 13 paralogs, with four of them belonging to group I, which mediate the conjugation of jasmonate and salicylic acid, while the others mediate the conjugation of IAA (Terol et al., 2006; Fu et al., 2011b). A recent study of the IAA biosynthesis mutant showed that auxin activity is linked with seed longevity in Arabidopsis (Pellizzaro et al., 2020). Auxin is also involved in regulating seed maturation and storability through stimulating abscisic acid (ABA) signaling (Carranco et al., 2010; Liu et al., 2013; Pellizzaro et al., 2020). ABA plays a critical role in seed desiccation tolerance, germination and storability through complex signaling networks (Rajjou et al., 2012). A bZIP transcriptional factor *ABA-INSENSITIVE3* (*ABI3*) is a central ABA signaling component in regulating seed maturation, desiccation tolerance, germination, and storability (Sugliani et al., 2009; Delahaie et al., 2013). *ABI3* could directly regulate the LATE EMBRYOGENESIS ABUNDANT (*LEA*) protein genes. *ABI3* also activated *HEAT SHOCK FACTOR A9* (*HSF A9*), a seed-specific transcription factor, to increase the accumulation of heat shock proteins (HSPs) and enhance seed storability (Kotak et al., 2007). Seed storability is gradually acquired during seed maturation, accompanied by accumulations of protective molecules, such as nonreducing sugars, LEAs, and HSPs, after the acquisition of desiccation tolerance (Tejedor-Cano et al., 2010; Hundertmark et al., 2011; Li et al., 2017). LEAs are small hydrophilic, largely unstructured, and thermostable proteins that accumulate during seed maturation (Zinsmeister et al., 2016) and have various protective functions, including antioxidant activity, hydration buffering, and membrane and protein stabilization (Delahaie et al., 2013), thus playing pivotal roles in modulating seed storability. However, few QTLs were colocalized with the genes related to the protective substances or antioxidants in plants. The roles of auxin in regulating antioxidants have not been fully explored, and the genetic basis of seed storability in rice remains largely unknown.

In the present study, two common major QTLs for seed storability were identified by combining GWAS and linkage mapping analyses in rice chromosome substitution segment lines (CSSLs) and a panel of rice germplasms under different natural storage and artificial aging (AA) conditions. Of them, *qSS1* was validated and further cloned through a map-based cloning approach. Transgenic experiments indicate that *OsGH3-2* encoding an IAA-amido synthetase is the gene underlying *qSS1*. *OsGH3-2* conjugates IAA to amino acids, forming inactive amide-linked auxin to regulate endogenous IAA levels. Moreover, *OsGH3-2* inhibited ABA signaling and reduced the accumulation of LEAs, leading to poor seed storability. These findings suggest that auxin acts as a key factor in modulating seed storability and *OsGH3-2* can be used as a promising gene to improve seed storability in rice.

## Results

### Natural variation of seed storability in rice

**Figure 1:**
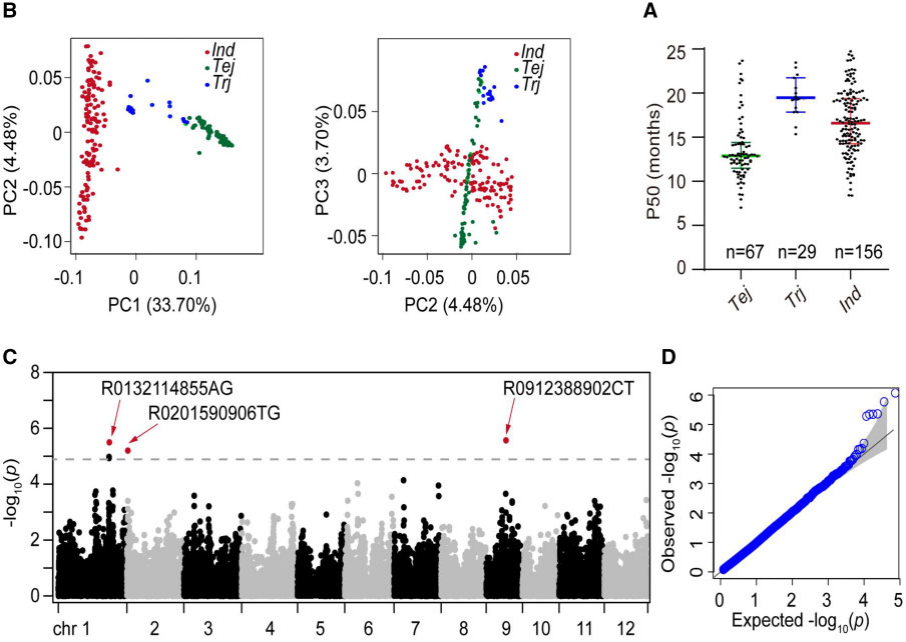
Variation in seed storability in a panel of rice germplasm. A, Difference in seed storability assayed by P50 (the time for germination percentage to decrease to 50%) among three subgroups. The edges represent the range of the 25th to 75th percentiles with the mean value shown by a bold middle line. *Ind*, *indica*; *Tej*, temperate *japonica*; *Trj*, tropical *japonica*, *n* represents the number of accessions. B, PC analysis of 252 rice accessions classifying three rice subgroups based on the high-density SNP markers. The proportion of variance explained by the first three PCs is indicated in the axis labels. C, Manhattan plot depicting significant SNPs associated with seed storability in the rice germplasm panel. The *x*-axis represents SNP locations across 12 chromosomes, and the *y*-axis indicates the −log_10_ (*P*-value) of the SNP association. The leading peaks are highlighted. The dotted line indicates the significance threshold after correction for multiple testing. D, Quantile–Quantile plot for the mixed-linear model analysis

To elucidate the genetic basis of the natural variation of seed storability in rice germplasm, the seed germination percentage of 252 rice accessions (Supplemental Data Set S1) was measured at the freshly harvested stage and every three months after harvest during 24 months of storage under warehouse conditions (65% RH, 25°C). Germination assays showed a gradual decrease in the germination percentage of most accessions after 6-month storage and near loss of seed viability at 24-month storage (Supplemental Figure S1). However, some varieties maintained a germination percentage above 50% even at 24-month storage under the warehouse conditions. The seed storability estimated by the P50 value (the time for germination percentage to decrease to 50%) from germination curves of six indicated times (6–24 months) during storage exhibited a large variation in these varieties (Figure 1A).

To identify the loci for the seed storability variation via GWAS of the rice germplasm, the accessions were genotyped using a high-density SNP array (RiceSNP50) as described previously (Chen et al., 2014a). The high-quality SNPs divide the accessions into three main subgroups. Three top principal components (PCs) explained 41.9% of the genetic variance within the panel (Figure 1B). The P50 value was significantly different among the three subgroups (Figure 1A). The average value of P50 of the temperate *japonica* (*Tej*) subgroup was 13.0 months, less than those of *indica* (*Ind*; 16.6 months) and tropical *japonica* (*Trj*) varieties (18.5 months; Figure 1B). This finding is consistent with previous results where *Ind* varieties exhibited stronger seed storability than temperate *japonica* (Miura et al., 2002). Genome-wide analysis with the mixed-linear model integrating the first three PCs as covariates identified three significant leading SNPs for seed storability assayed by P50 (Figure 1, C and D). Among them, the peak SNP on chromosome 1 was also localized within the QTL region (*qSS1*) in the *indica*/*japonica* CSSL population as described below.

### QTLs of seed storability in CSSLs

**Figure 2:**
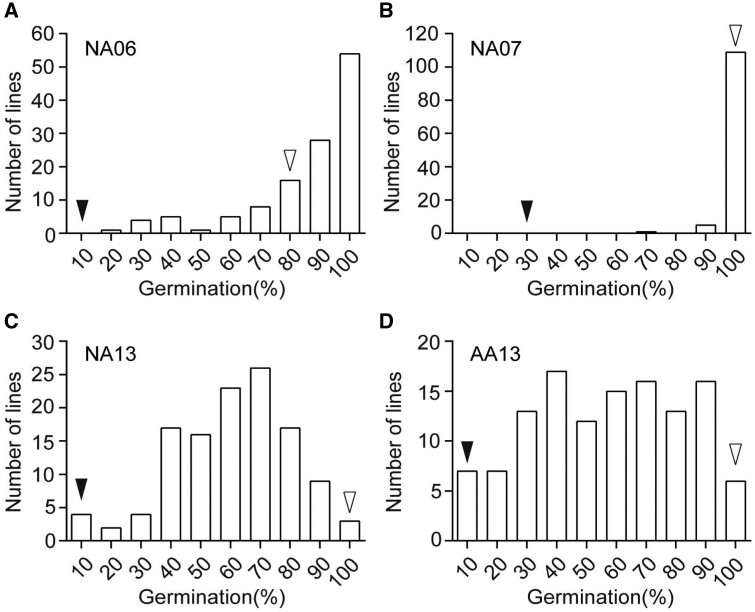
Frequency distribution of seed germination percentage of the 9311/NIP CSSL population. Germination percentage of the seeds produced in 2006 (A), 2007 (B), and 2013 (C), under NA. The seeds produced in 2013 (D) under AA. Black and white arrowheads indicate the average seed germination percentage of NIP and 9311, respectively

To further identify the loci associated with seed storability, the CSSL population derived from a cross of an *Ind* cultivar 9,311 with strong seed storability and a *Tej* cultivar Nipponbare (NIP) with weak seed storability was developed as described previously (Xu et al., 2007; Yuan et al., 2019). Seeds of the CSSL population were produced in 2006, 2007, and 2013 and were subsequently stored for various times or in natural aging (NA) conditions (65%RH, 25°C; Supplemental Figure S2A). The CSSL population seeds generated under three production environments and stored for 1 year under natural storage conditions were called as NA06, NA07, and NA13. In addition, the seeds of the CSSLs produced in 2013 were treated by AA (43°C and 88% RH) after 3 months of storage and were named AA13. Germination tests showed wide variations of the germination percentage with continuous distribution in the same CSSL population of seeds that were produced in different years (Figure 2), indicating that seed storability is a quantitative trait controlled by multiple genes. The germination percentages exhibited significantly positive correlations among these populations treated with NA and AA, except for NA6 and AA13 (Supplemental Figure S2B).

**Figure 3:**
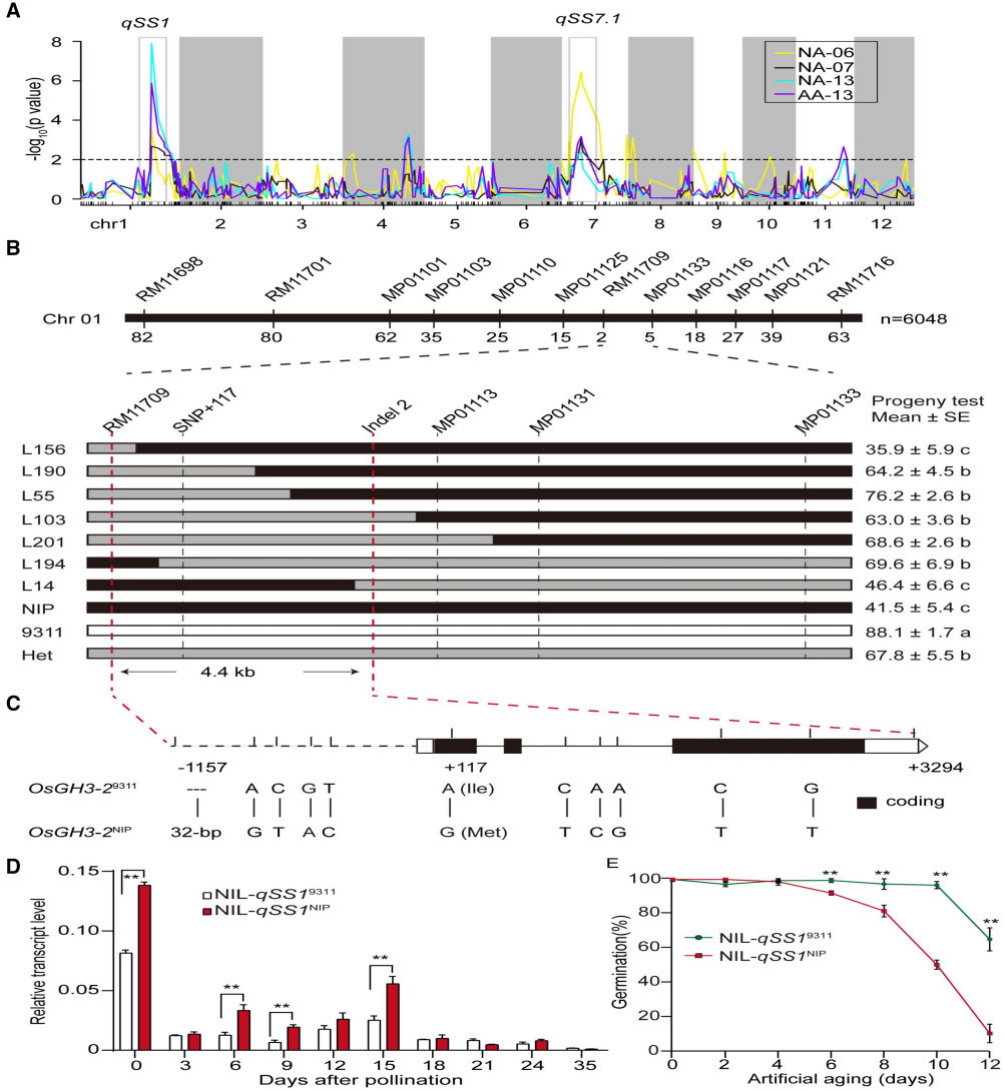
Map-based cloning of *qSS1*. A, QTL mapping of seed storability in the CSSLs under four natural storage and AA conditions. The *x*-axis represents bins along each numbered chromosome, which are separated by white and gray colors; the *y*-axis represents the −log_10_ (*P-*value) for the significant association. The dotted line indicates a threshold of *P *=* *0.01 for the QTL declaration. Two major loci detected repeatedly are highlighted. B, Fine mapping of *qSS1*. Graphic genotypes of the recombinants are indicated with germination percentages by progeny test. The means ± standard error (*n *=* *10) are provided, different letters denote significant differences at *P *<* *0.05 according to LSD test. C, Schematic gene structure of *OsGH3-2* showing relative positions of SNPs between NIP and 9311. Deleted nucleotides are depicted by dashes. The nonsynonymous SNP with the amino-acid change is indicated. D, Relative transcript levels of *OsGH3-2* between NIL-*qSS1*^9311^ and NIL-*qSS1*^NIP^ developing seeds at indicated days after pollination. The data are relative to the geometric average of the two reference genes, and the means ± standard error (of three biological replicates) are provided. Asterisks indicate significant differences between NILs at *P *<* *0.01 by Student’s *t* test. E, Germination assay of the seeds of NIL-*qSS1*^9311^ and NIL-*qSS1*^NIP^ under AA (88%RH, 43°C) on different days. Germination percentage is provided by the means ± standard error (of three biological replicates). Asterisks indicate significant differences (*P *<* *0.01) between NILs by Student’s *t* test

QTLs for seed storability by assessing the P50 values were analyzed using the linear ridge regression model in the CSSLs. A total of 19 QTLs were identified under NA and AA conditions with a threshold of *P *<* *0.01 (Supplemental Table S1). Nine, two and four QTLs of seed storability were identified with the phenotypic variance explained (PVE) of 58.9%, 17.3%, and 42.7% under NA06, NA07, and NA13, respectively. The alleles from 9311 at all loci, except *qSS7.2* and *qSS11*, increased seed storability. Four loci were detected explaining 40.3% of PVE in AA13. Among the 19 detected loci, 2 QTLs as major loci were identified in common in the CSSL population under all natural storage and AA conditions, including *qSS1* located in Bin027 with PVE of 7.4%–24.8%, and *qSS7.1* mapped in Bin 226 with PVE of 5.2%–20.2%. In addition, *qSS4.2* and *qSS11* were identified repeatedly in the same population under two storage conditions, and the remaining QTLs were identified under only one specific condition (Figure 3A;  Supplemental Table S1). These results suggest that seed storability is controlled by both major and minor effect genes and influenced by environments of harvest and storage.

### *OsGH3-2* is the gene underlying *qSS1*

In a previous study, *qSS1* was also identified in backcross inbred lines derived for a cross of the common parents 9311 and NIP (Yuan et al., 2019). Therefore, *qSS1* was a priority target to be cloned and characterized. Fine-mapping was initially conducted on a F_2_ population (composed of 6,048 individuals) from the cross of near-isogenic line (NIL)-*qSS1*^NIP^ and 9311, and 145 recombinant plants were selected with the markers of RM11698 and RM11716. Based on progeny testing of the recombination lines and genotyping with 14 additional markers in the target interval, *qSS1* was delimited to a 4.4-kb region, containing only one predicted gene, *OsGH3-2*, that encodes an IAA-amido synthetase (Figure 3B). Sequence comparison of this 4.4-kb region revealed one SNP in the first exon of *OsGH3-2*, which caused a predicted amino acid change from Methionine (Met) in NIP to Isoleucine (Ile) in 9311. In the promoter region, one insertion/deletion (Indel) and four SNPs were found between NIP and 9311 (Figure 3C). *OsGH3-2* was expressed in almost all rice organs but preferentially expressed in the developing seed and the root at the three-leaf seedling stage (Supplemental Figure S3A), which is consistent with the previous expression pattern investigated in many tissues or organs covering the entire life cycle of rice (Wang et al., 2010). In addition, *OsGH3-2* was differentially expressed in the developing seeds between NILs at 0, 6, 9, and 15 d after pollination (DAP), with an approximate 2.5-fold higher expression level in NIL-*qSS1*^NIP^ (weak seed storability) than in NIL-*qSS1*^9311^ (strong seed storability; Figure 3D). Furthermore, the seeds of paired NILs that carry the contrasting alleles of 9311 and NIP at *qSS1* in the same background of 9311 showed a significant difference in storability. NIL-*qSS1*^NIP^ was more sensitive to AA and had lower seed storability than NIL-*qSS1*^9311^ (Figure 3E). Therefore, *OsGH3-2* is the most likely candidate gene responsible for *qSS1*.

**Figure 4:**
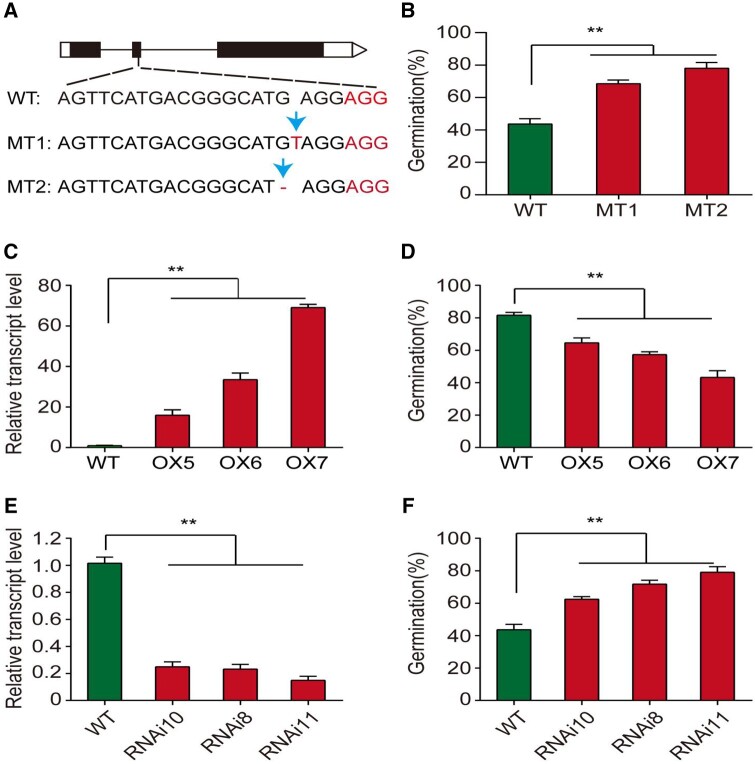
Transgenic experiments determine the effect of *OsGH3-2* on seed storability. A, Schematic gene model of *OsGH3-2* showing the partial sequence alignment of two independent homozygous mutants (MT1 and MT2) and wild-type (WT) at the target site. The nucleotide differences among WT and MT and the protospacer adjacent motif (AGG) are marked. B, Germination percentage of the seeds of WT and CRISPR-induced *OsGH3-2* mutants (MT) under AA conditions (88% RH, 43°C, 8 d). C, Relative transcript levels of the OX lines overexpressing *OsGH3-2* in the developing seeds at 6 d after pollination. D, Germination percentage of the seeds of the WT and OX lines under AA conditions. E, Relative transcript levels of *OsGH3-2* relative to *UBIQUITIN* in developing seeds of the WT and *OsGH3-2*-RNAi lines at 6 d after pollination. F, Germination percentage of the seeds of the WT and RNAi lines under AA conditions. The data are provided with the means ± standard error (*n *=* *3). Asterisks indicate significant differences compared with WT at *P *<* *0.01 by Student’s *t* test

To analyze the function of *OsGH3-2* on seed storability, transgenic experiments, including CRISPR-induced, overexpression, and RNAi, were conducted in rice. First, knockout mutants of *OsGH3-2* were generated by the CRISPR-induced approach. Two homozygous knockout *OsGH3-2* mutants (KOs) were obtained and evaluated for seed storability. One with one-base deletion in the second exon (as in mutant MT2), which caused a frame-shift variant producing a truncated peptide, while another with one-base insertion (as in mutant MT1) caused a premature stop codon, also leading to a truncated peptide (Figure 4A). Both homozygous KO mutants had a higher germination percentage than wild-type (WT) after AA (Fig. 4B). Next, the overexpression construct containing the entire *OsGH3-2* gene of 9311 was generated and introduced into the *japonica* variety ZH11, which had similar weak seed storability as NIP. Compared with the WT, three homozygous transgenic lines overexpressing *OsGH3-2*^9311^ (OX) decreased seed storability in a dose-dependent manner (Figure 4, C and D). Consistently, the knockdown of *OsGH3-2*^ZH11^ by RNAi showed increased seed storability compared with the WT (Figure 4, E and F), suggesting that both *Ind* and *Tej* alleles of *OsGH3-2* function in seed storability. Taken together, these results indicate that *OsGH3-2* is the gene responsible for *qSS1* and negatively regulates seed storability at the transcriptional level.

### *OsGH3-2* altered endogenous IAA and repressed ABA and LEA levels

**Figure 5:**
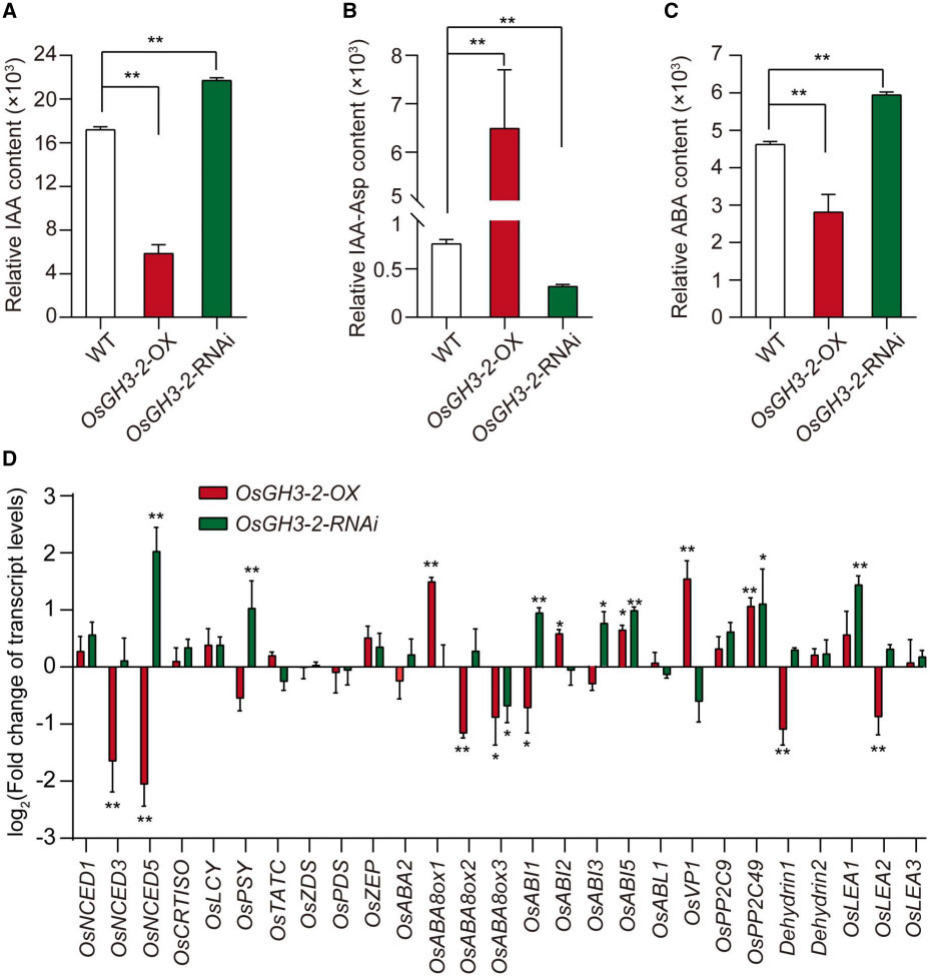
*OsGH3-2* modulates IAA and ABA contents. IAA (A), IAA-Asp (B), and ABA (C) in the mature seeds of *OsGH3-2* overexpression lines (OX), RNAi lines (RNAi), and wild-type (WT; *n *=* *6). D, Fold change of relative transcript level of the key genes related to ABA biosynthesis, catabolism and signaling and LEAs proteins in the transgenic lines in developing seeds at 6 d after pollination. The transcript levels of each assayed gene are relative to the geometric average of the two reference genes. The error bars represent standard error (*n *=* *8). Asterisks (*, **) indicate significant differences compared with WT at *P *<* *0.05 and *P *<* *0.01 by Student’s *t* test, respectively

*OsGH3-2* can catalyze the IAA conjugation to amino acids to removes excess free IAA and regulates homeostasis of endogenous IAA in rice (Du et al., 2012). To determine whether *OsGH3-2* has the IAA conjugation ability influencing endogenous IAA levels, a high-performance liquid chromatography-mass spectrometry (HPLC-MS) system was used to measure the contents of endogenous IAA and IAA-Asp in the mature seeds of transgenic lines. The amount of IAA was significantly reduced, while the IAA-Asp content was increased in the OX lines compared with those in WT (ZH11). *OsGH3-2*-RNAi (KD) and KO lines had lower IAA-Asp and higher IAA contents in the mature seeds than the WT (Figure. 5, A and B; Supplemental Figure S4). The results revealed that *OsGH3-2* conjugates IAA to Asp, resulting in the reduction of endogenous IAA. This finding is consistent with a previous report that the conjugation of free IAA to IAA-Asp led to IAA degradation in Arabidopsis (Westfall et al., 2016). In addition, *OsGH3-2* expression was rapidly induced by exogenous IAA treatment (Supplemental Figure S3B). This induction of *OsGH3-2* expression could provide an effective strategy to prevent over-accumulation of free IAA in rice. These data confirmed that *OsGH3-2* has IAA-amido synthetase activity and regulates the formation of IAA-Asp conjugates leading to homeostasis of endogenous IAA in rice.

IAA plays a critical role in regulating seed maturation and dormancy through stimulating ABA signaling (Liu et al., 2013). To investigate whether *OsGH3-2* affects the ABA pathway, the endogenous ABA contents in the mature seeds of the transgenic lines were measured. HPLC-MS quantification of ABA revealed that relative endogenous ABA contents in the OX lines were markedly decreased by at least 1.2-fold, while significantly increased by 1.5-fold in the KD lines (Figure 5C) compared with the WT. Consistent with the relative ABA levels, the expressions of several key genes associated with ABA biosynthesis, catabolism and signaling were significantly altered in the developing seeds at 6 DAP of these transgenic lines. Among them, many genes were of interest because they were regulated in the OX and KD of *OsGH3-2* lines in an opposite manner. In particular, *OsNCED5*, which encodes 9-*cis*-epoxycarotenoid dioxygenase as a key enzyme in ABA biosynthesis (Huang et al., 2019), was significantly repressed in the OX lines and significantly induced in the KD lines (Figure 5D). In addition, *OsABA8ox1*, a key enzyme that oxidizes ABA to phaseic acid (Saika et al., 2007), was significantly activated in the OX lines but did not change in the KD lines (Figure 5D). Therefore, high expression of *OsGH3-2* inhibits ABA biosynthesis and induces ABA metabolism in the developing seeds, leading to low ABA content. As expected, the expression levels of the ABA signaling genes, such as *OsABI1* and *OsABI3*, were downregulated in the OX lines and significantly upregulated in the KD lines (Figure 5D).

*ABI3* directly regulates *LEA* genes that play pivotal roles in acquiring desiccation tolerance and modulating seed longevity in Arabidopsis (Hundertmark et al., 2011). Intriguingly, the relative transcript levels of *Dehydrin1* and *OsLEA2* were upregulated in the KD lines, while significantly downregulated in the OX lines (Figure 5D), of which more accumulation of LEAs could enhance seed storability. Consistent with the expression of the *LEA* genes, the KD lines exhibited strong seed storability compared with the WT. Hence, these results suggest that *OsGH3-2* modulates the ABA pathway to inhibit the accumulation of *LEAs*, resulting in seed storability variation in rice.

### Variation in *OsGH3-2* is associated with seed storability

**Figure 6:**
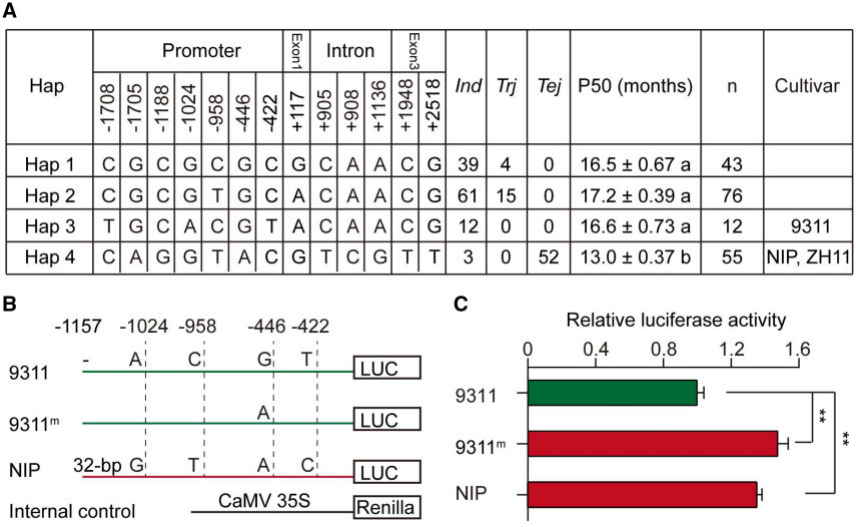
Differences in seed storability (P50) among haplotypes of *OsGH3-2*. A, Four haplotypes in a panel of rice germplasm. The positions of SNPs are indicated for the haplotype analysis. *Ind*, *indica*; *Tej*, temperate *japonica*; *Trj*, tropical *japonica*. n, number of varieties. The means ± standard error are given for each haplotype. The different letters denote significant differences according to LSD test at alpha = 0.05. B and C, Transient expression test of *OsGH3-2* in rice protoplasts*.* B, Model of the tested promoter segments of 9311 and NIP in which the SNPs are indicated. 9311^m^ represents the promoter with a mutated variant at SNP^−446^. C, Transcriptional activities of *OsGH3-2* driven by the NIP, 9311 or 9311^m^ promoter. The relative activity of firefly luciferase (LUC) to rLUC is given as the means ± standard error (*n *=* *6). Renilla luciferase (rLUC) was introduced simultaneously as an internal control. Asterisks indicate significance at *P *<* *0.01 by Student’s *t* test

Sequence comparison revealed that there are five variants in the promoter region and one nonsynonymous SNP^A117G^ in the coding region of *OsGH3-2* between NIP and 9311. The SNP^A117G^ was predicted to cause an amino-acid substitution (Lle to Met; Figure 3C). To determine whether the variants are associated with seed storability in a wide rice germplasm, 186 out of 252 accessions were re-sequenced at the gene region (Chen et al., 2014b). There were 13 nucleotide variations of *OsGH3-2* between NIP and 9,311 in the rice germplasm. Based on these SNPs, the 186 accessions were categorized into four haplotypes (Figure 6A). Hap4, including the representative cultivars NIP and ZH11, was predominant in temperate *japonica* cultivars, while Hapl, and Hap3, including the cultivar 9311, were dominant in *indica* subspecies. Hap4 had the lowest seed storability, exhibiting a significant divergence from the other three haplotypes (Figure 6A). Consistent with the different transcript level of *OsGH3-2* between NIL-*OsGH3-2*^9311^ and NIL-*OsGH3-2*^NIP^ (Figure 3D), transient expression assays in rice protoplasts demonstrated that the relative activity of *luciferase* driven by the NIP promoter was significantly increased compared with that by the 9,311 promoter (*P *<* *0.01; Figure 6, B and C). In addition, the relative activity of *luciferase* driven by the 9,311 promoter harboring one site mutated (G to A) at SNP^−446^ exhibited significantly higher level than that of the 9311 promoter. These results suggest that SNP^−446^ could be the functional variant accounting for the expression difference between the *OsGH3-2* alleles and provide additional evidence demonstrating that *OsGH3-2* regulates seed storability at the transcriptional level.

## Discussion

Seed storability has received wide attention as it is a critical characteristic of seed quality and an important adaptive trait. However, it is difficult to map and clone the genes associated with this complex trait, which is affected by the environment during seed formation, harvest, and storage (Finch-Savage and Bassel, 2015; Hay et al., 2018). In the present study, we used the common CSSL population generated from a cross of NIP (temperate *japonica*) and 9,311 (*indica*) that was grown in different harvest environments and evaluated seed storability under both natural storage and AA conditions. Two major loci (*qSS1* and *qSS7.1*) were consistently detected in the CSSLs under various harvest and storage conditions (Figure 3A;  Supplemental Table S1). *qSS1* is colocalized in the same or overlapping regions in previous studies (Miura et al., 2002; Hang et al., 2015; Liu et al., 2018; Yuan et al., 2019). In parallel, three leading SNP regions were identified for seed storability in a panel of rice germplasms (Figure 1C), which was assayed by the P50 value from germination curves, rather than germination percentage at a given time point during 24-m storage. Notably, the peak SNP detected on chromosome 1 was also located in *qSS1.* Hence, *qSS1* for seed storability identified in different populations under variable harvest and storage conditions is robust and worthy of subsequent study. The map-based cloning strategy with CSSL-derived segregating population identified *OsGH3-2* for seed storability *qSS1* and found that inactivation of IAA by conjugation is associated with seed storability variation in rice. The expression level variation of *OsGH3-2* largely caused seed storability difference between NIP and 9311, in which *OsGH3-2* was expressed at a low level that is associated with a strong storability phenotype.

*OsGH3-2* reportedly inhibits the accumulation of free IAA, resulting in higher resistance to pathogen invasion (Fu et al., 2011a; Sauer et al., 2013) and drought stress (Du et al., 2012) in rice. In addition, overexpression of *OsGH3-2* and its homologs, such as *OsGH3-1* and *OsGH3-8*, led to enhanced resistance to pathogens (Ding et al., 2008; Domingo et al., 2009; Zhang et al., 2009). However, it has yet to be reported whether *OsGH3-2* is related to seed storability. In this study, we found that the *OsGH3-2* KD and KO mutants exhibited significantly increased seed storability and the OX lines significantly decreased seed storability compared with the WT (Figure 4). Our data provide new information on the roles of *OsGH3-2* in the regulation of seed storability.

One notable finding is that *OsGH3-2* modulates seed storability involving the ABA biosynthesis and signaling pathway (Figure 5, C and D). We found that *OsNCED5* was repressed in the OX plants overexpressing *OsGH3-2*, while it was induced in the KD plants suppressing *OsGH3-2*. On the other hand, *OsABA8ox1*, which triggers ABA catabolism, was upregulated in the OX plants. Therefore, the ABA content was decreased significantly in the OX plants, but increased in the KD plants compared with those in the WT plants. Furthermore, many ABA signaling-related genes, such as *OsABI1* and *OsABI3*, were upregulated in the KD lines, and downregulated in the OX lines. These results are consistent with the fact that auxin acts upstream of ABA and activates the genes related to ABA signaling. *ABI3* plays a role downstream of the auxin signaling in Arabidopsis. The *abi3* mutants were corresponded with reduced dormancy, intolerance to desiccation and rapid viability loss during dry storage (Nambara et al 1992; Ooms et al, 1993; Clerkx et al., 2003, 2004a). In the present study, *OsGH3-2* negatively regulated seed storability, and its enhanced IAA-amino conjugation activity reduced *ABI3* transcript level. In addition, the expression level of *OsGH3-2* was rapidly induced to the highest level at approximately 2 h and declined after exogenous IAA treatment. In parallel to this response of *OsGH3-2* to IAA, *OsABI3* was also induced at approximately 4 h after IAA treatment (Supplemental Figure S3). These results suggest that *OsGH3-2* and *ABI3* are inducible by auxin, which links auxin and ABA responses in seeds. Intriguingly, the LEA-related genes are possible downstream targets of *ABI3*. Previous studies have reported that LEA proteins (e.g. dehydrin) progressively decreased in Arabidopsis seeds as AA time increased, and germination ability was lost, suggesting its involvement in seed longevity (Rajjou and Debeaujon, 2008). We found that *Dehydrin1* and *OsLEA2* were upregulated in KD lines and significantly downregulated in the OX plants (Figure 5D). Consistent with the results in Arabidopsis (Hundertmark et al., 2011), downregulation of the *LEA* genes decreased seed storability. Therefore, we propose that *OsGH3-2* modulates seed storability through catalyzing IAA conjugates with Asp to inactivate IAA, which is involved in the ABA biosynthesis and signaling pathway and probably subsequently altered protective proteins such as LEAs. In addition, *OsGH3-2* is expressed at a bimodal pattern with a peak around 15 DAP during seed development (Figure 3D), which coincides with major accumulation of IAA and correspond with the initiation of starch and storage protein synthesis and the establishment of seed dormancy (Basunia and Nonhebel, 2019). In line with this, significant differences were observed in seed length, 1,000-grain weight and seed dormancy between the KO mutants and WT (Supplemental Figure S5). These data indicate the gene also plays a role in seed development, but the functions of the gene on seed development warrant investigation in the future.

While *OsGH3-2* is responsible for the peak SNP on chromosome 1, some possible candidate genes within an approximately 150-kb region covering the other two peak SNPs associated with seed storability could be exploited. The first peak SNP region on chromosome 2 contains a HSP20 family gene (*Os02g03570*) and a pyridoxal phosphate synthase gene (*OsPDX2*). *OsPDX2* is essential for the biosynthesis of pyridoxine, which has antioxidant properties in plants (Mangel et al., 2019). The second peak SNP region on chromosome 9 harbors three annotated genes: *OsTPP7*, *OsTPS8*, and *OsHSPB1*. The former two are related to trehalose-6-phosphate metabolism, of which *OsTPP7* catalyzes the dephosphorylation of trehalose-6-phosphate to trehalose, which protects proteins and membranes from degradation (Paul et al., 2008). *OsTPP7* was suggested to be the candidate gene of *qLG-9* for seed storability in a previous study (Sasaki et al., 2015). In addition, *OsHSPB1* encodes a heat shock factor binding protein gene related to HSP. Thus, these genes associated with protective substances might be promising candidates associated with seed storability and warrant further investigation. In conclusion, the cloning and characterization of *OsGH3-2* for *qSS1* along with the elucidation of its natural allelic variation provide insight into the IAA and ABA metabolism associated with seed storability in rice and facilitate the improvement of rice tolerance to biotic or abiotic stresses during plant growth, development, and seed storage through the use of gene editing or genomic tools.

## Materials and methods

**CSSL population and rice germplasm.** A rice (*Oryza sativa*) CSSL population consisting of 120 lines was developed using a marker-assisted backcross scheme, with each line carrying a single or a few particular chromosome segments from *japonica* cultivar NIP that were introduced into *indica* variety 9311 (Xu et al., 2007). To determine the genotypes precisely, the CSSLs were re-analyzed using an Infinium RICE6K array (Illumina, San Diego, USA). The chip hybridization, SNP calling, genotyping, and map construction of the CSSL population were conducted as described previously (Yu et al., 2014). The bin was defined by a unique overlapping substitution segment over the CSSLs. A total of 357 bins were constructed based on the SNP genotypes (Zhang et al., 2020). A pair of NILs (NIL-*qSS1*^NIP^ and NIL-*qSS1*^9311^) carrying the contrasting alleles of NIP and 9311 at *qSS1* within the common background of 9311 was generated using a marker-assisted backcross scheme (Yuan et al., 2019).

A panel of rice germplasm comprised of 156 *indica* cultivars, 67 temperate *japonica* cultivars and 29 tropical *japonica* cultivars was also used in this study (Supplemental Data Set S1; Sun et al., 2017). Their genotypes were analyzed using the RiceSNP50 array as described previously (Chen et al., 2014a). A total of 41,850 SNPs was detected after filtering with a minor allele frequency less than 1% and missing data less than 30%. The positions of SNPs were referenced to the rice genome assembly MSU6.1 (http://rice.plantbiology.msu.edu/). In addition, to analyze the haplotypes of *OsGH3-2*, 186 accessions from the germplasm panel were re-sequenced at the target sites as previously described (Chen et al., 2014b).

**Measurement of seed storability under different treatments.** The CSSL population was grown at the experimental field of Huazhong Agricultural University in Wuhan, China (30.48°N, 114.2°E) in 2006, 2007, and 2013 (Supplemental Figure S2A). Bulked seeds from each line were harvested at 35 d after flowering and equilibrated in a storage chamber with a low relative humility (RH, 25%) for one month to obtain a constant moisture content of approximately 12%. After equilibration, the seeds of CSSLs produced in 2006 and 2007 were stored at the ambient conditions with 25°C and 65% RH for 1 year, then stored at –20°C for 4 or 5 years. Seed deterioration occurred during this process at the above conditions with 25°C and 65% RH termed as NA. Therefore, these two batches of seeds produced in 2006 and 2007 were named NA06 and NA07, respectively. The seeds of the CSSLs produced in 2013 were stored at moderately accelerated aging conditions (75% RH, 25°C) for one month and divided into two subsets for different treatments. After equalibration to a constant moisture content, one subset of the seeds (named NA13) were stored at the warehouse conditions (25°C and 65% RH) for 1 year, followed by germination tests to assess seed viability. Another subset of the seeds was exposed to AA (88% RH, 43°C). Thus, the population under AA was called AA13. The AA was conducted as described in a previous study (Yuan et al., 2019), in which seeds were treated at a high temperature (43°C) and RH (88%) for 8 d in a thermostatic moisture regulator (HWS-080, Shanghai, China). The seeds of each treated population were germinated as previously described (Yuan et al., 2019), and the germination percentage was used to determine the degree of seed storability.

To assess seed storability of rice germplasm, a germination test was performed after storage for 0, 6, 9, 12, 15, 18, 21, and 24 months under warehouse storage conditions (25°C, RH 65%). From the survival curves of germination at the indicated time points from 6- to 24-month storage, seed storability represented by P50 was determined using Sigmoidal Equation with GERMINATOR software (Joosen et al., 2010).

**QTL analysis.** QTL analysis was performed as described previously (Sun et al., 2015). In brief, a ridge linear regression analysis was performed to detect QTLs in the CSSL population using the “ridge” (http://www.r-project.org/) with the ridge parameters chosen automatically from R package. A significance level of *P *<* *0.01 was set as the threshold to declare the presence of a putative QTL in a given bin. If several adjacent bins showed significant *P-*values, the QTL was tentatively located in the most significant bin. The variance explained by each QTL (bin) was calculated using the function “*lmg*” from R package.

**Genome-wide association analysis.** PC analysis was performed using the GCTA software (version 1.26.0; Yang et al., 2011). The first three eigenvectors were retained to create a plot in two or three dimensions. GAPIT (version 2) was used for GWAS analysis with a mixed-linear model using the first three principle components as co-factors (Tang et al., 2016). To determine the genome-wide threshold for significant marker-trait association, Bonferroni correction was used (Li et al., 2012). The genome-wide threshold was *P *=* *10^−5^ after the corrections.

**Map-based cloning.** DNA was extracted from young seedling leaves using the cetyltrimethylammonium bromide method (Murray and Thompson, 1980). Simple sequence repeat markers were designed according to the website Gramene (http://www.gramene.org/), and the new Indel markers were developed according to the sequence variations between NIP and 9311. The seed storability of the recombinant lines was determined by progeny test with 10 individuals for each line. The target genes from NIP and 9311 were sequenced via the BigDye Terminator Cycle Sequencing v3.1 (Applied Biosystems, Foster, USA). The primers used for fine-mapping and sequencing are listed in Supplemental Data Set S2.

**Vector construction and rice transformation.** Three types of transgenic experiments were conducted to determine the effects of *OsGH3-2* on seed storability. To construct the overexpression vector, the *OsGH3-2*-coding region from 9311 was cloned into the linearized pCAMBIA1301s driven by the CaMV 35S promoter (Zhou et al., 2009). For RNAi, a 513-bp fragment of *OsGH3-2* cDNA was cloned into the linearized pDS1301 with *Kpn*I-*Sac*I restriction sites (Chu et al., 2006). The CRISPR/Cas9 vector was constructed following a previously described method (Sun et al., 2016). All constructs were confirmed by sequencing, introduced into *Agrobacterium tumefaciens* strain EHA105, and transferred into the rice variety ZH11 (Hiei et al., 1994). All primers used for the transgenic experiments are listed in Supplemental Data Set S2.

**Expression analysis.** Seeds were freshly harvested at different developmental stages and directly frozen in liquid nitrogen. Total RNA was extracted using a TRIzol Reagent Kit (Invitrogen, Carlsbad, USA). Real-time quantitative PCR was conducted using SYBR Green Master (Roche Diagnostics, Mannheim, Germany) with the ABI 7500 Real-Time PCR System. The rice *UBIQUITIN* (*UBQ*) and *β-TUBULIN* genes were used as the internal control, and expression levels of the assayed genes relative to the geometric average of the two reference genes were analyzed as previously described (Livak and Schmittgen, 2001; Vandesompele et al, 2002). Experiments were performed on three independent biological replicates from pools of 200 seeds harvested on three plants and on three technical replicates. Specific primers for expression analyses are listed in Supplemental Data Set S2.

**Quantification of endogenous phytohormones and conjugates.** A liquid chromatography-electrospray ionization-tandem mass spectrometry method was used for the relative quantification of endogenous phytohormones (ABA and IAA) and IAA conjugates (Chen et al., 2013). Briefly, mature seeds (100 mg for each replicate) were frozen in liquid nitrogen, ground to a fine powder, and extracted twice with 750 μL 80% methanol solution (methanol:water:acetic acid, 80:19:1, v/v/v), vigorously shaken on a shaking bed for 16 h at 4°C in the dark, and then centrifuged at 13,000 rpm for 15 min at 4°C. The supernatants were filtered using a syringe-facilitated 13-mm diameter nylon filter with pore size 0.22 μm (Nylon 66; Jinteng Experiment Equipment Co., Ltd, Tianjing, China). The filtrate was dried under nitrogen gas for approximately 4 h at room temperature, and then dissolved in 200-μL methanol. Quantification was performed in an ABI 4000 Q-Trap LC-MS system (Applied Biosystems, CA, USA). The relative signal intensities of endogenous phytohormones were normalized by first dividing them by the intensities of the internal standard (lidocaine, 0.1 mg L^−1^) and then log_2_ transformation to improve the normality. Six replicates of each seed sample were measured for phytohormone quantification.

**Transcriptional activation analysis.** The promoters of *OsGH3-2* from NIP, 9311 and the mutants were cloned into pGREEN0800 with *Kpn*I to drive the luciferase reporter, respectively. The CaMV 35S promoter-driven *Renilla* was used as an internal control. Rice protoplast isolation and transformation were based on the method described previously (Zhang et al., 2011). The Dual-Luciferase^R^ Reporter Assay System (Promega) was used to analyze the transcriptional activation of the promoter with a Tecan Spark 10 M microplate reader (Tecan Group Ltd., Zurich, Switzerland). Each sample was measured with six biological replications.

### Accession numbers

Sequence data from this article can be found on the Rice Genome Annotation Project website (http://rice.plantbiology.msu.edu/) under the following accession numbers: *OsGH3-2* (LOC_Os01g55940), *OsNCED1* (LOC_Os02g47510), *OsNCED3* (LOC_Os03g44380), *OsNCED5* (LOC_Os12g42280), *OsCRTISO* (LOC_Os11g36440), *OsLCY* (LOC_Os02g09750), *OsPSY* (LOC_Os09g38320), *OsTATC* (LOC_Os01g31680), *OsZDS* (LOC_Os07g10490), *OsPDS* (LOC_Os03g08570), *OsZEP* (LOC_Os04g37619), *OsABA2* (LOC_Os03g59610), *OsABA8ox1* (LOC_Os02g47470), *OsABA8ox2* (LOC_Os08g36860), *OsABA8ox3* (LOC_Os09g28390), *OsABI1* (LOC_Os05g49730), *OsABI2* (LOC_Os05g51510), *OsABI3* (LOC_Os01g68370), *OsABI5* (LOC_Os01g64000), *OsABL1* (LOC_Os06g10880), *OsVP1* (LOC_Os06g43660), *OsPP2C9* (LOC_Os01g62760), *OsPP2C49* (LOC_Os05g38290), *Dehydrin1* (LOC_Os01g50700), *Dehydrin2* (LOC_Os11g26760), *OsLEA1* (LOC_Os01g50910), *OsLEA2* (LOC_Os04g49980), *OsLEA3* (LOC_Os05g46480), *β-TUBULIN* (LOC_Os01g59150).

## Supplemental data

The following materials are available in the online version of this article.

**Supplemental Figure S1.** Seed viability of rice germplasms during 24 m of storage.

**Supplemental Figure S2.** Correlation of seed storability of the 9311/NIP CSSLs in different experiments.

**Supplemental Figure S3.** Expression patterns of *OsGH3-2* in various rice tissues and responses of *OsGH3-2* and *OsABI3* to IAA treatment.

**Supplemental Figure S4.** *OsGH3-2* affects IAA and ABA contents in mutants.

**Supplemental Figure S5.** Differences in the seed-related traits between the CRISPR-induced mutants (MT) and wild-type (WT).

**Supplemental Table S1.** QTLs for seed storability identified in the 9311/NIP CSSLs.

**Supplemental Data Set S1.** List of 252 rice accessions used in this study.

**Supplemental Data Set S2.** Primers used in this study.

## Supplementary Material

kiab059_Supplementary_Data

## Notes

S.Y. conceived and designed the experiments; Z.Y. performed most of the experiments; K.F. performed the transgenic experiments; Y.W., L.T., and C.Z. participated in the fine mapping and phenotyping; C.Z., W.S., and H.H. analyzed the data; Z.Y. and S.Y wrote the article. All authors read and approved the final manuscript.

The author responsible for distribution of materials integral to the findings presented in this article in accordance with the policy described in the Instructions for Authors (https://academic.oup.com/plphys/pages/general-instructions) is: Sibin Yu (ysb@mail.hzau.edu.cn).
