# Supplementary material for: OsGRETCHENHAGEN3-2 modulates rice seed storability via accumulation of abscisic acid and protective substances
Source: Plant Physiol. 2021 Feb 11;186(1):469–82. doi: 10.1093/plphys/kiab059 (PMC8154041; doi:10.1093/plphys/kiab059)
Supplement: kiab059_Supplementary_Data [file kiab059_supplementary_data.zip › pp.01355.2020-s01.pdf]

OsGRETCHENHAGEN3-2 **modulates rice seed storability via accumulation of abscisic acid and protective substances**

Zhiyang Yuan, Kai Fan, Yuntong Wang, Li Tian, Chaopu Zhang, Wenqiang Sun, Hanzi He, Sibin Yu*

*Corresponding author: Sibin Yu (ysb@mail.hzau.edu.cn)

Supplemental Figures S1-S5 and Table S1

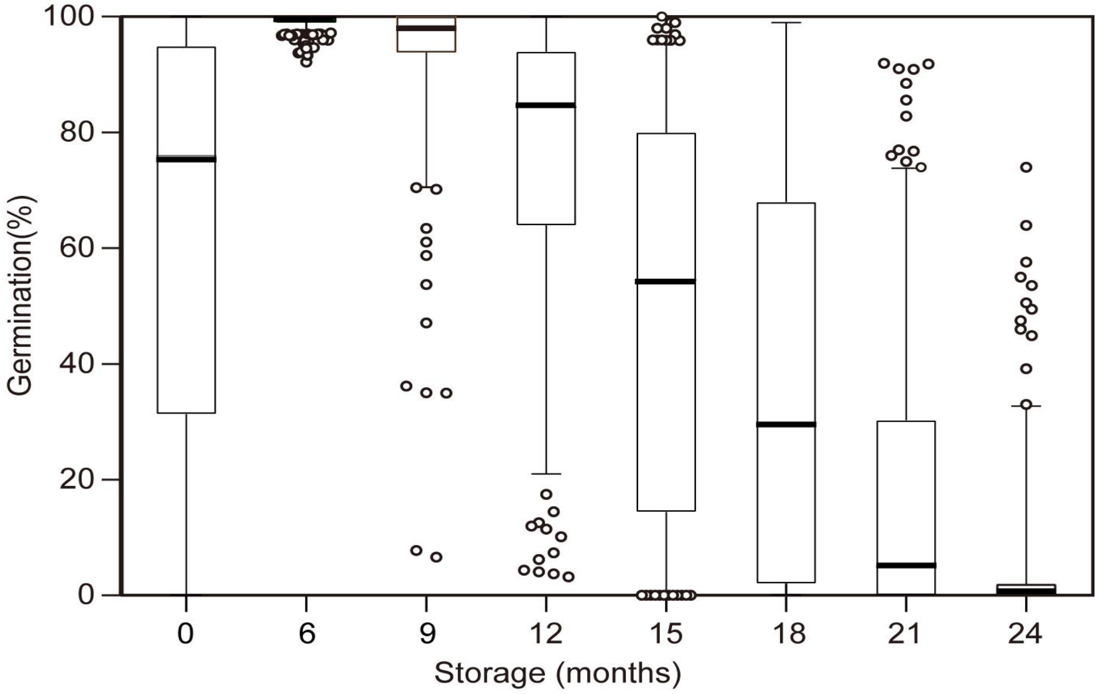

Supplemental Figure S1. Seed viability of rice germplasms during 24 m of storage. The box edges represent the range of the 25th to 75th percentile with the median value shown by a bold middle line. Whiskers represent 1.5 times the quantiles of the data and open dots are outliers (*n* =252).

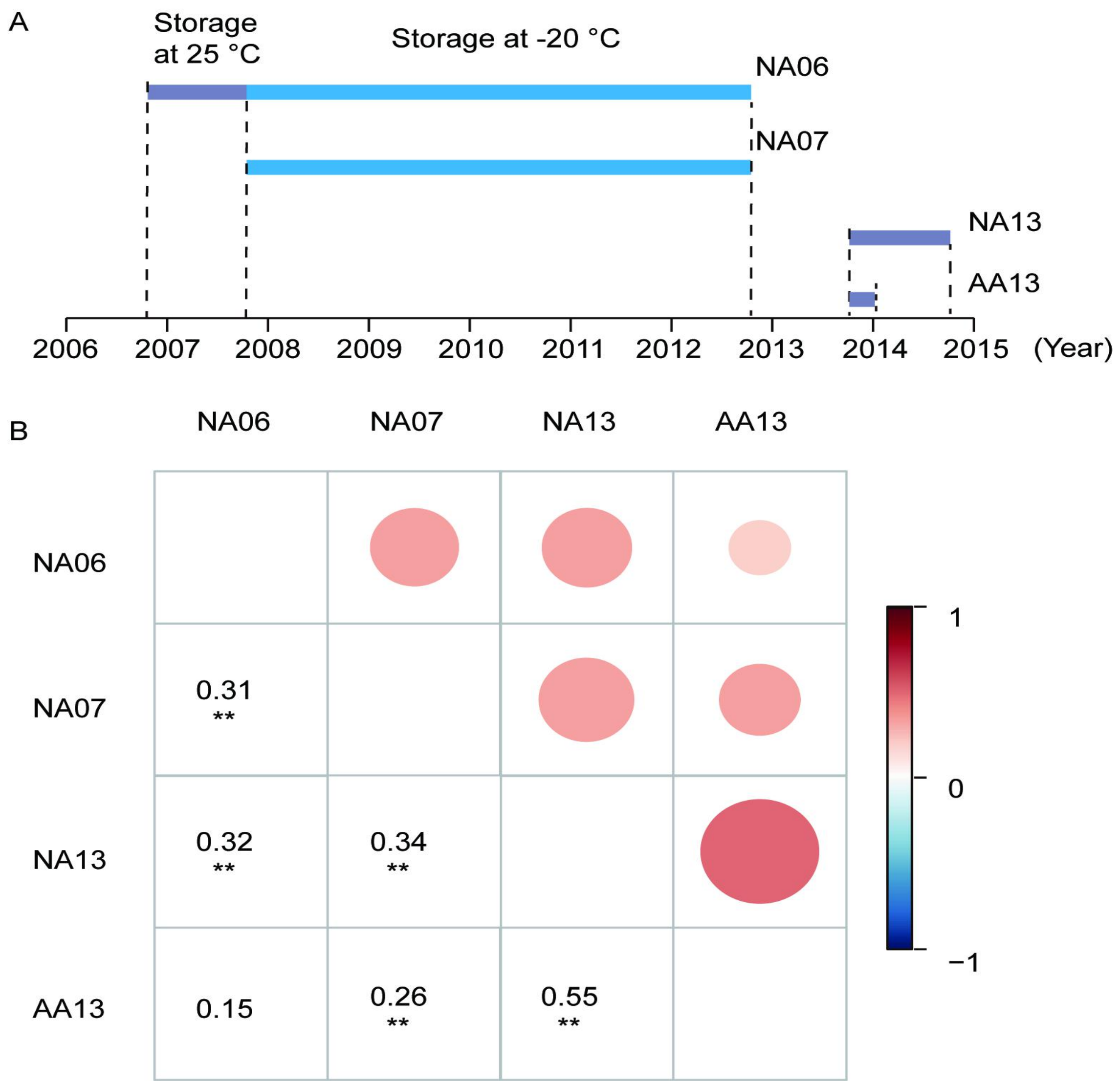

Supplemental Figure S2 Correlation of seed storability of the 9311/NIP CSSLs in different experiments. **A,** Timeline of seed production and storage experiments of the CSSLs produced in 2006, 2007, and 2013. **B,** Heatmap of Pearson's correlation coefficients between seed storability (lower left triangle) of the CSSLs under natural storage (NA) and artificial ageing (AA) conditions. Asterisks indicate significance at $p < 0.01$ by Student's t-test ($n = 120$).

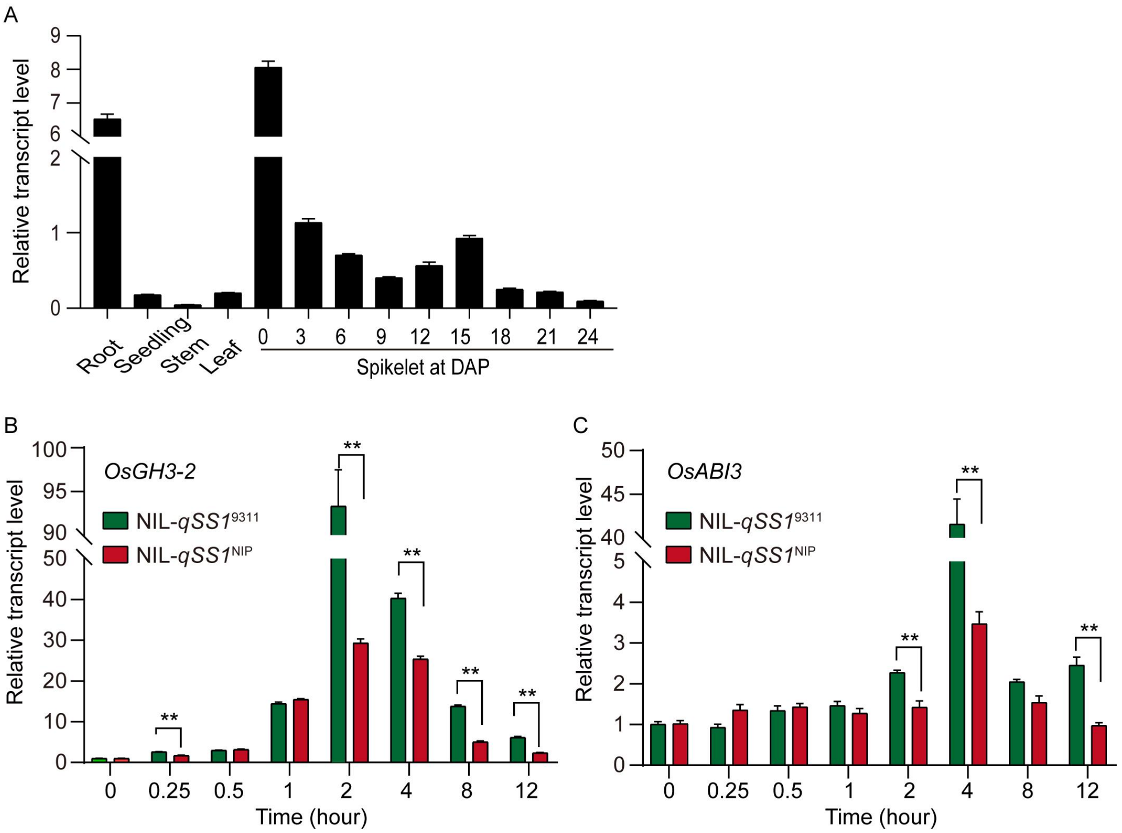

Supplementa1 Fig. S3 Expression patterns of *OsGH3-2* in various rice tissues and responses of *OsGH3-2* and *OsABI3* to IAA treatment. **A**, Relative transcript levels of *OsGH3-2* relative to *UBIQUITIN* in various tissues. Root and shoot at the three-leaf seedling stage; stem and leaf at the flowering stage; and developing spikelets at indicated day after pollination (DAP). Data are the means ± standard error (with three biological replicates). Expression of *OsGH3-2* (**B**) and *OsABI3* (**C**) of the seedling shoot of NILs at the three-leaf stage under 100 μM IAA treatment. Data are the means ± standard error of six replicates. Asterisks indicate significant differences at p <0.01 between NILs by Student's t-test.

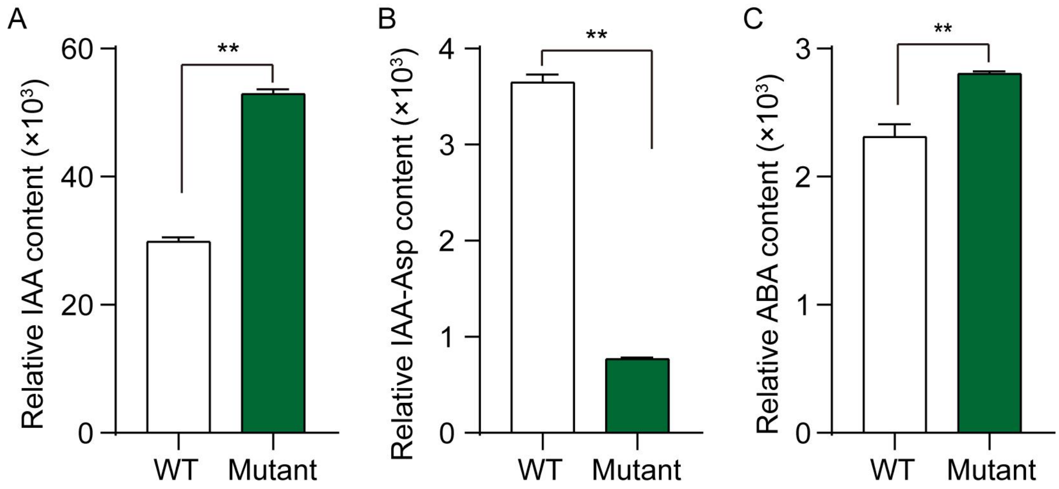

Supplemental Figure S4 *OsGH3-2* modulates IAA and ABA contents. IAA (**A**), IAA-Asp (**B**), and ABA (**C**) in mature seeds of the CRISPR-induced mutants and wild type (WT). Asterisks indicate significant differences between the mutant and wild type (WT) at $p < 0.01$ by Student's *t*-test. Data are the means ± standard error ($n = 6$).

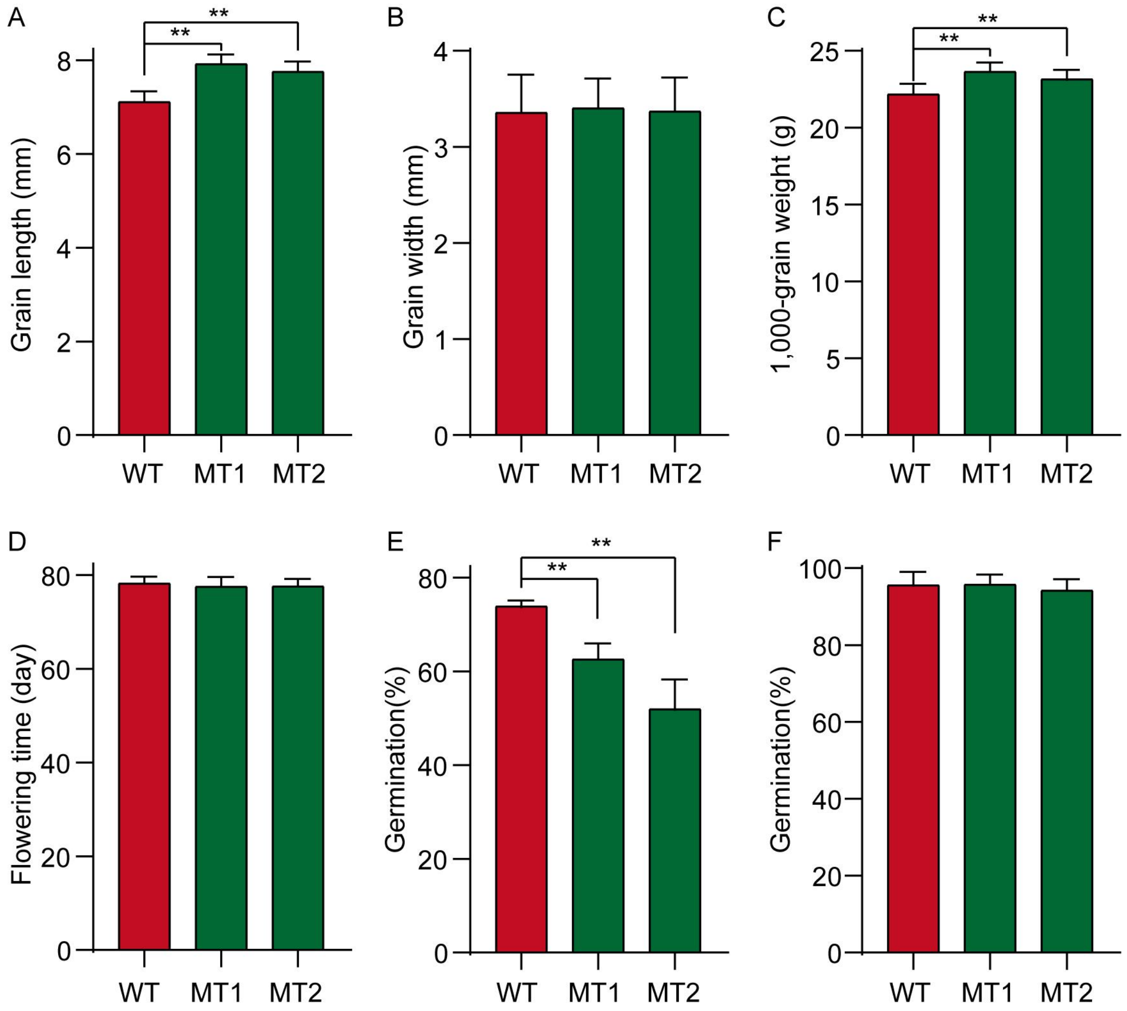

Supplemental Figure S5. Differences in the seed-related traits between the CRISPR-induced mutants (MT) and wild type (WT). **A**, Grain length. **B**, Grain width. **C**, 1,000-grain weight. **D**, Flowering time. **E**, Seed dormancy, measured by germination percentage of seeds harvested at 35 DAP. **F**, Germination percentage after break seed dormancy (under 50°C,7 days). Data in A-D represent means ± standard error (*n* = 30); Data in E-F represent means ± standard error (*n* = 3). Asterisks denote significant difference at $p < 0.01$ by Student's *t*-test.

Supplemental Table S1. QTLs for seed storability identified in the 9311/NIP CSSLs.

Traits[a]	QTL[b]	Chr.	Bin	Interval (Mb)[c]	Add[d]	*p* value	PVE (%)
NA06	*qSS1*	1	Bin027	30.36-33.27	-0.043	3.04E-04	7.42
	qSS4.1	4	Bin131	4.41-4.83	-0.028	4.92E-03	5.77
	qSS4.2	4	Bin159	31.80-32.90	-0.020	5.68E-03	3.73
	qSS7.1	7	Bin226	8.63-9.29	-0.050	3.55E-07	20.23
	qSS7.2	7	Bin239	28.25-29.14	0.041	5.80E-04	5.31
	qSS8	8	Bin244	1.68-2.67	-0.030	7.82E-04	3.57
	qSS9.1	9	Bin263	0-2.65	-0.034	3.00E-03	4.61
	qSS9.2	9	Bin278	13.59-14.86	-0.057	4.46E-03	4.95
	qSS10	10	Bin291	11.57-13.27	-0.028	7.13E-03	3.30
NA07	*qSS1*	1	Bin027	30.36-33.27	-0.004	2.10E-03	8.07
	qSS7.1	7	Bin226	8.63-9.29	-0.002	9.76E-04	9.22
NA13	*qSS1*	1	Bin027	30.36-33.27	-0.056	1.30E-08	24.75
	qSS4.1	4	Bin151	28.09-28.25	-0.022	4.93E-04	4.53
	qSS7.1	7	Bin226	8.63-9.29	-0.023	4.33E-03	5.24
	qSS11	11	Bin333	20.74-22.15	0.031	9.50E-03	4.36
AA13	*qSS1*	1	Bin027	30.36-33.27	-0.061	1.33E-06	22.79
	qSS4.2	4	Bin151	28.09-28.25	-0.028	7.41E-04	5.35
	qSS7.1	7	Bin226	8.63-9.29	-0.036	6.74E-04	8.28
	qSS11	11	Bin333	20.74-22.15	0.040	2.25E-03	3.87

[a] NA06, NA07 and NA13 represent the seeds of the CSSL population produced in three years (2006, 2007 and 2013) and stored under natural conditions, respectively. AA13 represents the seeds of the CSSL population produced in 2013 and treated with artificial ageing.

[b] SS represents seed storability; Chr. Chromosome.

[c] Interval represents the genomic position for a given QTL based on MSU 6.1.

[d] Add and PVE (%) indicate additive effect and phenotypic variance explained by a given QTL, respectively.
